# Influence of fluid shear stress on human limbal epithelial cells

**DOI:** 10.1016/j.bbrep.2026.102453

**Published:** 2026-01-17

**Authors:** Sophia Masterton, Mark Ahearne

**Affiliations:** a Department of Mechanical, Manufacturing and Biomedical Engineering, School of Engineering, Trinity College Dublin, University of Dublin, Ireland; b Trinity Centre for Biomedical Engineering, Trinity Biomedical Sciences Institute, Trinity College Dublin, University of Dublin, Ireland

**Keywords:** Mechanobiology, Limbus, Corneal epithelium, Shear stress

## Abstract

**Background/aims:**

Limbal epithelial cells (LECs) have a crucial role in the maintenance of the corneal surface by migrating from the limbus to the cornea and replacing corneal epithelial cells. These cells are subjected to shear stress via blinking and tear film movement but the influence this stress has on the cells is poorly understood. The aim of this study was to examine how fluidic shear stress can affect the behaviour of LECs from different donors.

**Methods:**

A commercial fluid flow system (ibidi) was used to apply fluid shear at two different flow rates, with no flow being used as a control. Cells from three different donors were examined for phenotype, stratification, TRPV4 activation, cell adhesion and barrier function.

**Results:**

Both low and high shear stresses resulted in changes to the cell phenotype and these changes were highly donor dependent. Expression of TRPV4, a mechanosensitive ion-channel, was up-regulated for all donors exposed to shear stress. Stratification of cells only occurred with cells exposed to shear stress.

**Conclusion:**

This study show the importance of shear stress on modulating the behaviour of LECs and how donor to donor variations and the heterogeneity of cell populations need to be considered when conducting cell based studies.

## Highlights

• Fluid shear stress influences the phenotype and stratification of LECs.
• The effect of fluid shear stress is highly donor dependent.
• Dynamic culture conditions can be used to regulate LEC behaviour.

## 1 Introduction

The corneal epithelium is continually exposed to oscillatory shear stress generated by tear film dynamics and eyelid motion, a physiological process essential for maintaining corneal homeostasis, facilitating wound healing, and supporting lubrication and nutrient delivery. At the corneal periphery lies the limbus, a specialized region harbouring multiple cell types [1] including limbal epithelial stem cells responsible for regenerating the epithelial layer. Disruption of this regenerative capacity due to injury, chemical burns, or disease can lead to limbal stem cell deficiency (LSCD), a condition that may result in corneal opacity and blindness. The differentiation of cells into the corneal epithelial lineage is a highly regulated process, with recent transcriptomic analyses identifying thousands of genes differentially expressed during these stages [2]. Restoration of the limbal stem cell niche typically involves transplantation of autologous or donor-derived cells, either as cultured epithelial sheets or delivered using carrier scaffolds. However, several challenges hinder the efficacy of these approaches, including immunological rejection, reliance on animal-derived supplements such as fetal bovine serum (FBS), limited availability of basal stem cells, suboptimal culture conditions, and the use of non-approved biomaterials [[3], [4], [5]].

Despite the critical role of mechanical forces in ocular physiology, the precise magnitude and in vivo dynamics of shear stress acting on the corneal surface remain poorly characterized. To address this, prior studies have employed custom-designed bioreactor systems and organ-on-a-chip devices to simulate the mechanical environment of eye blinking, enabling investigation into drug toxicity and the mechanobiological responses of the corneal epithelium [6,7]. These in vitro models have also provided insights into how fluid shear influences epithelial differentiation and barrier integrity [8]. However, the shear stress rates of studies performed vary widely from 0.07 dyn/cm^2^ to 50 dyn/cm^2^ [8,9] as well as the length of time that the cells are exposed to shear with some studies exposing cells to shear for a couple of hours or seconds at particular days in culture. Data from previous studies has shown that oscillatory shear stress can enhance corneal epithelial cell differentiation and apoptosis, while also modulating migratory behaviour and wound healing capacity [10,11]. These findings underscore the importance of mechanical cues in corneal biology, although further work is needed to define physiologically relevant shear profiles and their downstream effects.

Although several studies report shear-dependent effects in corneal epithelia [12], none have examined how unidirectional laminar shear specifically affects human limbal epithelial cell (LEC) phenotype, TRPV4 expression, and stratification. The aim of this study was to examine how laminar fluid shear on human LECs effects phenotype, stratification and cell-cell adhesion. Using a commercially available perfusion system, LECs were exposed to either high (2.43 dyn/cm^2^) or low (1.1 dyn/cm^2^) levels of laminar shear stress for durations of 1 or 3 days. Gene expression analysis was conducted to quantify several markers associated with LECs, with the aim of elucidating the optimal shear conditions for LEC culture. Expression of the mechanosensitive ion channel TRPV4 was also evaluated to explore its potential involvement in mediating the cellular response to shear stress.

## 2 Materials and methods

### 2.1 Cell culture

Human corneal rims were obtained from the Royal Victoria Eye and Ear Hospital, Dublin after keratoplasty. All tissue used had written informed consent from the donor or next of kin for the tissue to be used for research purposes. Ethical approval for this study was provided by the Trinity College Dublin, University of Dublin, School of Medicine Research Ethics Committee (Ref: 20150607; Approved: June 17, 2015). In this study, cells were isolated from three donors of varying ages.

Culture media used in this study to obtain cells from tissue explants consisted of low glucose Dulbecco's Modified Eagle's Medium (DMEM) and Ham's F12 medium in a 3:1 ratio supplemented with 10 % Foetal Bovine Serum (FBS), 0.1 % penicillin/streptomycin solution, 1 % non-essential amino acids, 400 mM l-Glutamine, 6.8 mg 3,3,5-triiodo-l-thyronine sodium salt (T3), 1 μg/ml Insulin, 180 μM Adenine, 10 ng/ml Epidermal Growth Factor (EGF), 1 μg/ml isoproterenol, 0.4 μg/ml hydrocortisone and 5 μg/ml Transferrin.

To isolate LECs, 6-well plates were coated with gelatin (1 mg/ml) at 37 °C for 1 h. Any remaining gelatin was aspirated, and the plate left to dry 1 h. Each corneal rim was rinsed with PBS, quartered and placed epithelial side down onto the gelatin coated plates. For each explant to attach, 1 ml of media was placed on top of each explant and left overnight at 37 °C. The following day 2 ml media was then added to each explant culture. Media was replaced three times a week until the cells had grown out of the explant and occupied 80 % of the well surface area. Cells were released from the plates using accutase and transferred to T25 cell culture flasks for further expansion. Remaining explants from the initial expansion were fed again with corneal epithelial media and the process repeated to increase cell number which can be done up to three times [13]. Upon confluence in the T25 flask, cells were further expanded in a T75 flask and used for fluid flow experiments. All cells used in the experiment were at passage 4 or below.

### 2.2 Fluid flow bioreactor

A commercially available fluidic unit quad and pump (IBIDI) was used for shear stress experiments which allowed 8 samples to be run in parallel. μSlide I Leur 0.8 (IBIDI) slides were used for all experiments. The system operates by using a pump to generate constant pressure which moves media from one reservoir to another. This applied pressure results in a specific flow rate (ml/min) which depends on the channel height, pressure input, medium viscosity and flow resistance of the perfusion system. The flow rate (ml/min) results in a wall shear stress (dyn/cm^2^) to which the cells are exposed. The shear stress applied to the cells for this slide is calculated using the equation:

τ=34.7×η×ϕ

where τ is the shear stress, η is the fluid viscosity and ϕ is the flow rate. The constant air pressure from the pump to the reservoirs of the fluidic unit generates a constant flow of medium within the slides; before these reservoirs run dry the medium is pumped back and forth between the two reservoirs. To create a laminar flow two valves are integrated in the fluidic unit that are simultaneously switched between two states.

For our experiments, the slides were coated with gelatin for 1 h at 37 °C. LECs were then seeded at 500,000 cells/slide (200,000 cells/cm^2^) in 200 μl of media to allow a fully confluent layer of cells to be achieved. Cells were left to attach for 5 h and gently washed with media to remove any unattached cells. For the static cultures, cells were left in the slides with daily media changes for either 1 or 3 days. Cells undergoing flow were attached to the fluidic unit and subjected to high shear (2.43 dyn/cm^2^) or low shear (1.1 dyn/cm^2^) for either 1 or 3 days. After shear had been applied to cells for the appropriate amount of time cells were prepared for RT-PCR or fixed for immunocytochemical analysis.

### 2.3 RT-PCR

RT-PCR was performed using a similar protocol to those previously described [[14], [15], [16]]. RNA was isolated from monolayer cultures using Trizol (Invitrogen). The Trizol-cell solution was collected in RNAse-free eppendorf tubes, snap frozen in liquid nitrogen and transferred to −80 °C for storage until further use. When thawed, 200 μl of chloroform was added to each tube and centrifuged at 12,000 G at 4 °C. RNA located in the upper phase was transferred to a new RNAse free tube, isopropanol was added at the same volume as well as 4 μl glycoblue to allow visualisation of the RNA. The tubes were stored at −20 °C for 12 h, then placed on ice to allow the solution to thaw and centrifuged at 12000 G at 4 °C for 15 min. A visible blue RNA pellet was formed, supernatant was discarded and the tubes dried. 1 ml of 70 % ethanol (in RNAse free water) was added to wash the pellet. Another centrifugation step was performed at 12,000 G at 4 °C for 15 min, ethanol was removed and the pellet air-dried. RNAse free water (11 μl) was used to dissolve the pellet. A NanoDrop-1000 (Thermo Fisher Scientific) was used to quantify RNA yield and purity. Transcription of mRNA to cDNA was performed using a high-capacity cDNA reverse transcription kit (Invitrogen). A mastermix was added to 500 ng of RNA and placed in a thermocycler. The following temperature sequence was applied: 10 min at 25 °C, 2 h at 37 °C, 5 min at 85 °C, 1 min at 4 °C. Quantitative PCR was performed with TaqMan reagents, 4.5 μl cDNA, 5 μl TaqMan universal mastermix II and 0.5 μl primer. The following primers were used: NP63α (custom made primer sequence adapted from Robertson et al.) [17] ABCG2 (Hs01053790_m1), CK15 (Hs00267035_m1), Nestin (Hs00707120_s1), CK14 (Hs00265033_m1), CK3 (Hs00365080_m1), CK12 (Hs00165015_m1), TRPV4 (Hs01099348_m1) and GAPDH (Hs02758991_g1) housekeeping gene control. Fold change expression was calculated using the ΔΔCt method.

### 2.4 Immunocytochemistry

To perform immunocytochemical staining cells were seeded onto μslides I Leur 0.8 followed by PBS washing. After wither 1 or 3 days, cells were fixed using 4 % paraformaldehyde (PFA) for 15 min at room temperature followed by rinsing in phosphate buffered saline (PBS) 3 times and stored in PBS until ready to stain. Cells were blocked and permeabilised using 2 % FBS and 0.5 % Triton-X in PBS (blocking buffer) for 30 min. Cells were stained for cellular actin to visualise the cytoskeleton using Phalloidin-TRITC P1951 (Sigma Aldrich) at 1:1000 for 2 h at room temperature. All groups were also stained for cellular nuclei using fluoroshield with DAPI (Sigma Aldrich). The following antibodies and dilutions were used: Anti-NP63 at 1:500 dilution (619002 – Biolegend), Integrin β1 at 1:1000 dilution (ab24693 - Abcam), Vimentin at 1:1000 (ab92547 - Abcam), ZO-1 at 1:200 dilution (40–2200 - Biosciences). For integrin β1 goat anti-mouse IgG H&L (Alexa Fluor® 488) (ab150113 - Abcam) was used. For all other proteins donkey anti-rabbit IgG H&L (Alexa Fluor® 488) (ab150073 - Abcam) was used. Cells were imaged using a confocal microscope. Confocal z-stacks were acquired and representative optical sections were selected at comparable relative depths within the epithelial layer (apical, middle, or basal, depending on the marker analysed) to ensure consistent comparison between conditions.

### 2.5 Statistical analysis

All experiments were carried out in triplicate, statistical analysis and outlier calculation was performed using GraphPad Prism software. Data are presented as the mean ± standard deviation (SD), significance was calculated either via one-way or two-way ANOVA with Post-Tukey test, significance deemed as p ≤ 0.05 for all data sets.

## 3 Results

### 3.1 Cell phenotype

**Fig. 1:**
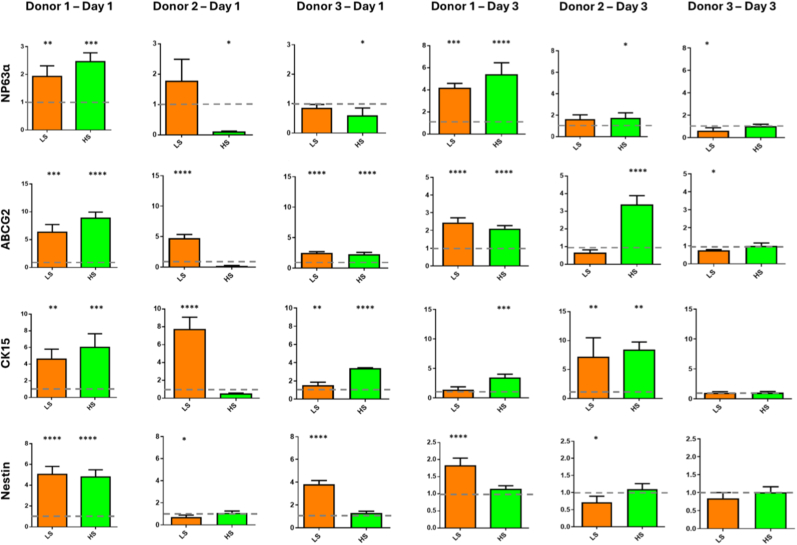
**Relative gene expression analysis of NP63α, ABCG2, CK15 and Nestin after 1 and 3 days of low shear (LS) and high shear (HS) culture for cells taken from 3 donors.** Dotted line represents the relative static control (value = 1). Data are presented as the mean (±SD), significance calculated via one-way ANOVA with a Post-Tukey test, N = 4, ∗ = P ≤ 0.05, ∗∗ = P ≤ 0.01, ∗∗∗ = P ≤ 0.001, ∗∗∗∗ = P ≤ 0.0001 compared to static control group.

Gene expression of LECs exposed to low and high shear rates were examined. Cells without shear force applied (static) were used as a control. A panel of markers associated with limbal cells (NP63α, ABCG2, CK15 and Nestin) were examined for cells isolated from 3 different donors after 1 and 3 days (Fig. 1). While the expression of most of these markers significantly increased for all 3 donors exposed to the low shear for 1 day, the magnitude of this increase varied between donors. For high shear, there were even great donor variations with donor 1 showing a significant increase in all markers compared to the static control, while donor 2 either showed no increase or a significant decrease in these markers and donor 3 showing increases in ABCG2 and CK15 and a decrease in NP63α. Similar donor variations were found after 3 days. Under low shear, there was a significant increase in NP63α, ABCG2 and Nestin for donor 1 and CK15 for donor 2 and significant decreases in Nestin for donor 2 and NP63α and ABCG2 for donor 3. Under high shear there was an increase in NP63α, ABCG2 and CK15 for donor 1 and 2 and no significant change in gene expression for donor 3 compared to the static control. Immunostaining for **NP63** was performed on Donor 2 cells to assess its **nuclear localization**, providing insight into the activation status of this marker (Fig. S1).

**Fig. 2:**
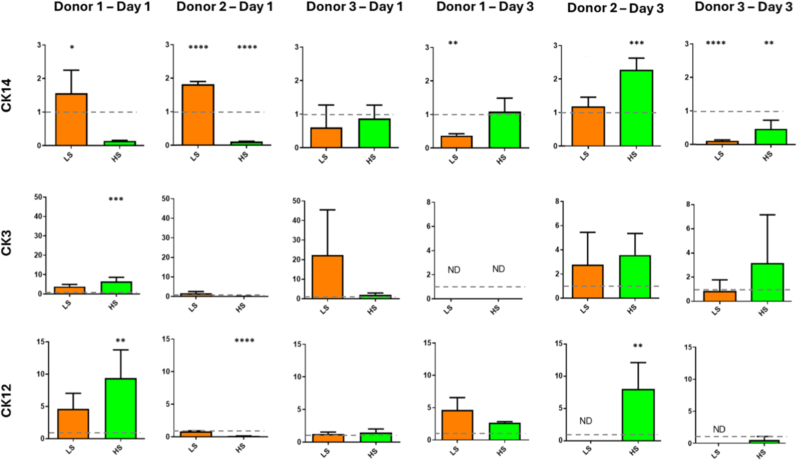
**Relative gene expression analysis of CK14, CK3 and CK12 after 1 and 3 days of low shear (LS) and high shear (HS) culture for cells taken from 3 donors.** Dotted line represented static control. Data are presented as the mean (±SD), significance calculated via one-way ANOVA with a Post-Tukey test, N = 4, ∗ = P ≤ 0.05, ∗∗ = P ≤ 0.01, ∗∗∗ = P ≤ 0.001, ∗∗∗∗ = P ≤ 0.0001 compared to static control group. ND = not detected.

The gene expression of transient amplifying marker CK14 and mature epithelial markers CK3 and CK12 were examined (Fig. 2). CK14 expression was significantly up regulated for just donor 2 after 1 day of low shear and down regulated for donors 1 and 2 after 1 day of high shear. After 3 days, CK14 was significantly down regulated for donors 1 and 3 under low shear conditions, down regulated for donor 3 under high shear and up regulated for donor 2 under high shear. After 1 day, CK3 and CK12 expression was up regulated under high shear for donor 1 and CK12 down regulated for donor 2. After day 3, there was no significant difference between groups although for several groups no gene expression was detected.

### 3.2 TRPV4 expression

**Fig. 3:**
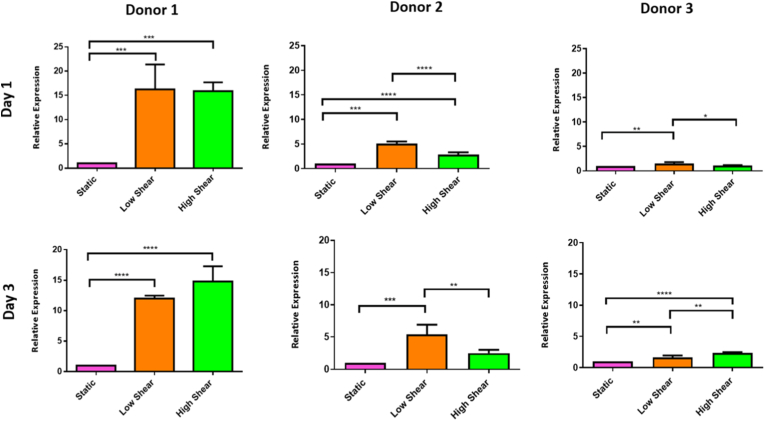
**Real time PCR of TRPV4 after 1 day and 3 days static, low shear and high shear culture.** Data are presented as the mean (±SD), significance calculated via one-way ANOVA with a Post-Tukey test, N = 4, ∗ = P ≤ 0.05, ∗∗ = P ≤ 0.01, ∗∗∗ = P ≤ 0.001, ∗∗∗∗ = P ≤ 0.0001.

Expression of TRPV4 was significantly upregulated in all donors under low shear culture after 1 and 3 days compared to static culture (Fig. 3). Donor 1 had a significant upregulation of TRPV4 after 1 day in both low and high shear conditions compared to the static control group. After 3 days the high shear group was more significantly upregulated compared to the static control group. Donor 2 had a significant upregulation of TRPV4 in the low shear stress cell culture after both 1 and 3, the high shear group also significantly increased TRPV4 expression compared to the static control group after 1 day. Donor 3 had the most significant upregulation of TRPV4 gene expression after 1 day in the low shear group compared to static control group; this was also significantly upregulated in the high shear. After 3 days of shear stress, TRPV4 was significantly upregulated in the high shear group in this donor compared to both the low shear and static groups in this donor. The low shear group also significantly enhanced TRPV4 gene expression after 3 days compared to the static control group.

### 3.3 Stratification

**Fig. 4:**
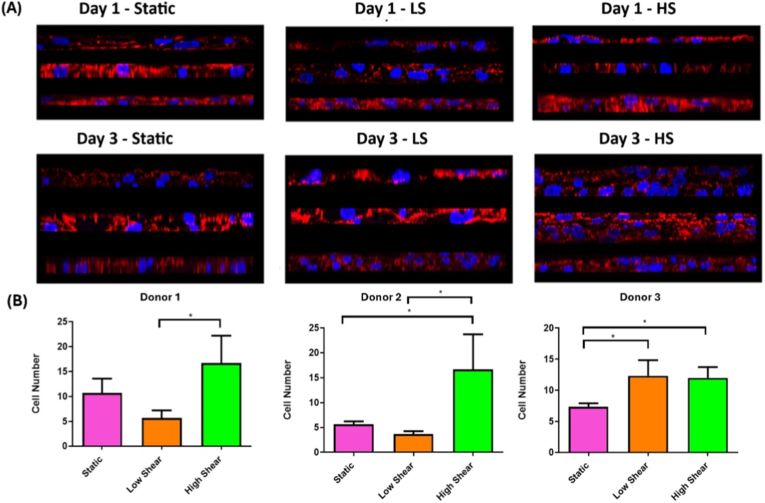
(A) Immunocytochemical staining for cellular stratification. Each image shows stratification from each donor (top = donor 1, middle = donor 2, bottom = donor 3) LS = low shear, HS = High shear. (B) Stratification of cells as a measure of cell number. Data are presented as the mean (±SD), significance calculated via one-way ANOVA with a Post-Tukey test, N = 3, ∗ = P ≤ 0.05.

Stratification of the cells under static and flow conditions was examined (Fig. 4A). All donors in the 1 day and 3 day static group remained as a monolayer with no stratification observed. Donor 3 stratified to form a 2–3 cell layer after 3 days of low shear stress with all other donors not showing any stratification. The high shear group after 1 day showed some stratification to a 2 cell layer in donor 3 but cells did not stratify in the other donors exposed to 1 day high shear. Cells exposed to 3 days of high shear stratified to form a 4–5 cell layer in both the donor 1 and 2 with a 2–3 cell layer observed after 3 day high shear in donor. This effect is further shown when comparing the maximum projection of cells from Donor 3 after 3 days in static and high shear cultures (Fig. S2).

Stratification was quantified by manually counting cell number using the Image J cell counter plugin on three separate orthogonal sections per donor in each condition. Cell number from each donor and shear stress group was quantified and these values used to calculate percentage increase of cell number using the static group as the control as shown in Fig. 4B. All donors displayed a significant increase in cell number after 3 days high shear stress compared to either static or low flow culture.

### 3.4 Cell adhesion

**Fig. 5:**
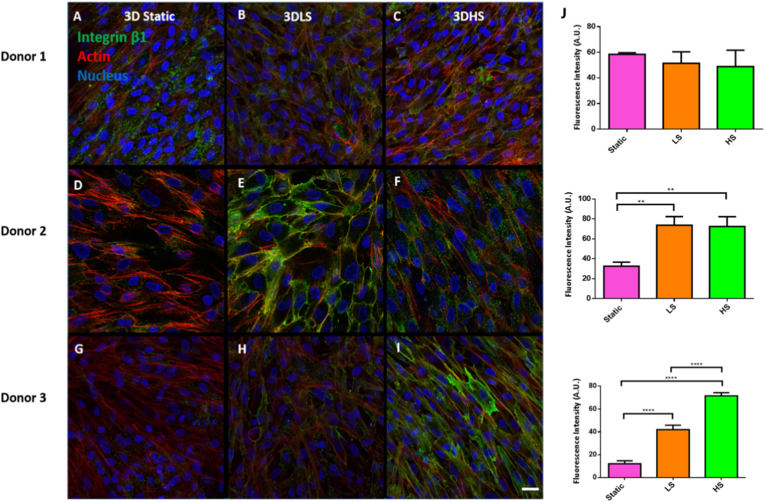
**Immunocytochemical staining for Integrin β1 (green) after 3 days static culture, low shear (3DLS) or high shear (3DHS) culture.** A – C Donor 1; D – F Donor 2; G – I Donor 3. All cells were counterstained with f-actin (red) and DAPI (blue), Scale bar = 20 μm. (J) Fluorescence intensity of Integrin β1. Data are presented as the mean (±SD), significance calculated via one-way ANOVA with a Post-Tukey test, N = 3, ∗∗ = P ≤ 0.01, ∗∗∗∗ = P ≤ 0.0001.

After 3 days donor 1 had a random distribution of integrin β1, donor 2 had an even distribution of integrin β1 in the shear groups but was more scattered in the static group and donor 3 had a scattered integrin β1 protein distribution in the static and low shear groups but was more evenly distributed in the high shear group. All donors displayed an elongated cell shape with the low shear group showing some more cobblestone morphology compared to the other groups (Fig. 5A–I). When integrin β1 fluorescence intensity was calculated, donor 1 has no significant change between groups while both donor 2 and 3 showed increased integrin β1 after shear stress (Fig. 5J).

**Fig. 6:**
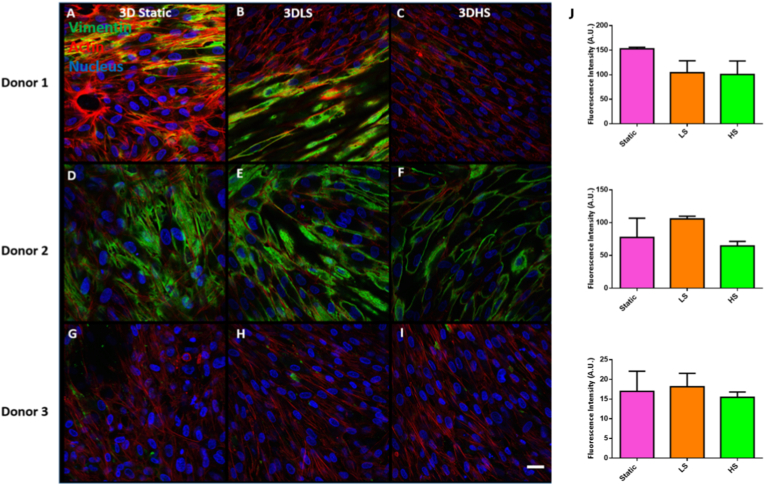
**Immunocytochemical staining for Vimentin (green) after 3 days static culture, low shear (3DLS) or high shear (3DHS) culture.** A – C Donor 1; D – F Donor 2; G – I Donor 3. All cells were counterstained with f-actin (red) and DAPI (blue), Scale bar = 20 μm. (J) Fluorescence intensity of vimentin. Data are presented as the mean (±SD), significance calculated via one-way ANOVA with a Post-Tukey test, N = 3.

Vimentin was distributed mostly at the top of the monolayer of cells. The images in the results below are from the middle of each stack (Fig. 6). Donor 1 had an even distribution of vimentin in the static and low shear group but showed random distribution of vimentin in some cells in the high shear group. Donor 2 had even distribution of vimentin across all groups. Donor 3 did not show much vimentin protein in this area of the stack, any protein that was visible was quite scattered. When vimentin fluorescence intensity was calculated, there was no significant differences in vimentin due to shear stress.

**Fig. 7:**
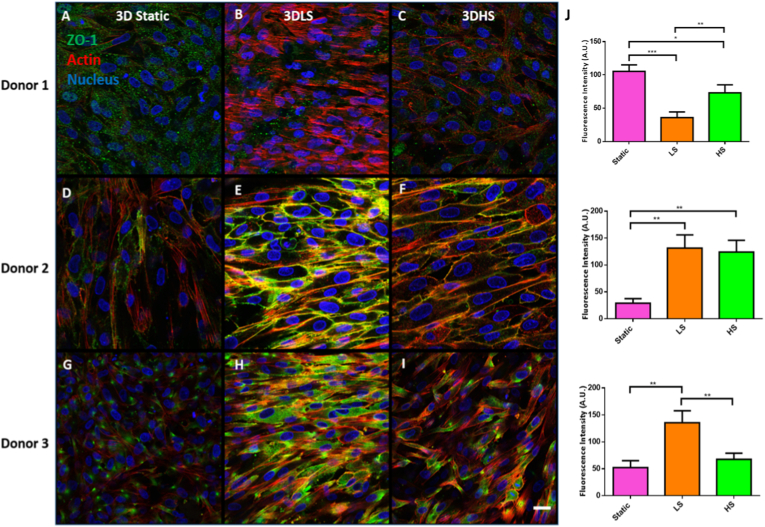
**Immunocytochemical staining for ZO-1 (green) after 3 days static culture, low shear (3DLS) or high shear (3DHS) culture.** A – C Donor 1; D – F Donor 2; G – I Donor 3. All cells were counterstained with f-actin (red) and DAPI (blue), Scale bar = 20 μm. (J) Fluorescence intensity of ZO-1. Data are presented as the mean (±SD), significance calculated via one-way ANOVA with a Post-Tukey test, N = 3, ∗ = P ≤ 0.05, ∗∗ = P ≤ 0.01, ∗∗∗ = P ≤ 0.001.

Confocal microscopy was used to determine if shear stress affected adherens junctions as shown in Fig. 7. ZO-1 protein was studied to determine expression and distribution of this protein under shear stress. Donor 1 had even distribution in the static and high shear group while the 3 day low shear group showed more sparse distribution of the protein. Donor 2 had less visible ZO-1 protein in the static group with this quite scattered in distribution. The low shear group showed an even ZO-1 distribution. This was also seen in the high shear group with less ZO-1 protein at each cell boundary. Donor 3 had even ZO-1 distribution among each group. This was most pronounced in the 3 day low shear group with the 3 day static and high shear group having more ZO-1 distribution at the apical cellular sides.

When ZO-1 intensity was quantified, donor 1 had a significant decrease in ZO-1 expression in both the low shear and high shear stress group compared to the static group. The high shear stress group significantly increased ZO-1 expression compared to the low shear group. Donors 2 and 3 significantly increased ZO-1 expression after 3 days of low shear stress compared to the static group. Donor 2 also had a significant increase in ZO-1 expression after 3 days of high shear stress compared to the static group. Donor 3 had a significant decrease in ZO-1 after high shear stress compared to the low shear stress group.

## 4 Discussion

A panel of markers were used to examine the response of LECs to shear stress. The markers selected range from stem cell associated markers expressed in the limbus, namely NP63α, ABCG2 and CK15 to the multipotent marker Nestin and the transient amplifying marker CK14. It should be noted that NP63 immunostaining has frequently been used qualitatively in limbal epithelial cell cultures as an indicator of an undifferentiated or progenitor-like epithelial phenotype, rather than to define stem cell frequency [18]. In addition, two mature epithelial markers CK3 and CK12 were examined. This allowed for a wide range of phenotypes to be examined. Unlike previous studies in the literature, this study used unidirectional rather than bidirectional oscillatory shear which has been used in the literature to mimic the conditions of eye blinking [6,8]. The aim of this study was not to mimic a more in-vivo phenotype of the corneal epithelium but rather elucidate what shear stress rate in a unidirectional manner would increase stem cell expression of the corneal cells and use this information to guide culturing conditions for expanding these cells ex-vivo for transplantation, something that has not been previously explored.

Among the shear stress environments examined, day 1 low shear showed the most significant upregulation of stem cell markers among donors which was decreased over three days. This suggests that exposing cells to day 1 of low shear can increase their expression of stem cell markers and that priming these cells to shear before culturing in static conditions may work to increase stem cell expression in the cells before using them for transplantation. There were observable differences in these results when comparing different donors, which may influence what shear stress should be used to increase stem cell marker expression depending on the age of the donor. Taking NP63α after 1 day as an example, donor 1 increased expression under both high shear and low shear, donor 2 only increased expression under low shear while donor 3 saw a decrease in expression under high shear. Therefore, donor age should be taken into consideration when using fluid flow to modulate stem cell marker expression. Immunostaining for NP63 was also performed to examine this effect at a cellular level. Notably, a greater proportion of cells exposed to shear exhibited nuclear NP63 compared with static cultures, suggesting that shear promotes nuclear translocation and activation of this stem cell marker. Other stem cell markers showed a similar **shear-dependent upregulation at the gene expression level**, although immunostaining was not performed for these proteins. Together, these data support and complement our gene expression results, reinforcing that shear stress enhances stem and progenitor marker expression in limbal-derived epithelial cells.

The transient amplifying marker CK14 was also significantly upregulated in response to 1 day of low shear in 2 out of 3 donors, however this response disappeared to was reverse by day 3. After 3 days of high shear stress this marker was significantly upregulated in donor 2 but down regulated in donor 3, again demonstrating the donor dependent nature of the cell's response. Mature markers CK3/12 were significantly upregulated under high shear in some donors but not low shear. After 3 days in some cases these markers were not detected. As no obvious trend was observed for these mature markers it cannot be concluded how shear affects mature markers of the corneal epithelium. However, coupled with increased stratification after 3 days of high shear this may suggest that a more in vivo like phenotype is observed with a heterogeneous population of stem cell and mature markers.

TRPV4 is a mechanosensitive ion channel implicated in MSC shear stress response [19] and has been shown to be activated by several different stimuli applied to corneal epithelial cells [20,21] but the effect on TRPV4 corneal epithelial cells by fluid shear stress has not previous been explored. In this study, it was shown that the TRPV4 gene was significantly upregulated in all donors in response to shear stress suggesting that TRPV4 may be involved in relaying mechanical signals in the fluid shear stress response. TRPV4 may serve as a therapeutic target in mimicking this shear stress response in vitro during cultivation of these cells for limbal stem cell transplantation.

Stratification of the cells after 3 days high shear coupled with a more mature phenotype suggests that this shear stress rate may be used to create a more in vivo like model of the corneal epithelium in vitro. It could also be used to cultivate cellular sheets for transplantation. Corneal epithelial cells which have differentiated to a mature phenotype have shown their capability to de-differentiate into stem cells to repopulate the stem cell niche when the limbus has been removed [22,23]. Therefore, exposing these cells to shear stresses may enable this functionality when transplanted into the eye.

Integrin proteins play a crucial role in maintaining the function of the stratified corneal epithelium with their impact depending on their localization, adhesive properties and expression levels [24]. Integrin β1 has been implicated in the wound healing capabilities of the corneal epithelium [25]. A previous study that looked at integrin β1 in corneal epithelial cells exposed to shear showed that integrin β1 was upregulated in cells exposed to shear suggesting an enhanced wound healing capability. In the presented study, shear stress increased integrin β1 expression in donor 2 and 3. Together with the observed CK14 expression, these findings indicate that shear stress can induce a pro-migratory phenotype in the corneal epithelium while maintaining expression of markers found in the limbus, making it a promising approach for ex-vivo cultivation.

The intermediate filament protein vimentin has been associated with an early differentiating highly motile phenotype of the corneal epithelium [26,27]. The effect that shear stress has on this protein has not been previously examined. No significant differences were observed after shear exposure, suggesting that shear stress does not affect vimentin expression. However, there was a large variation in vimentin staining between donors suggesting that this protein is highly donor specific.

ZO-1 was used to determine adherens junctions between cells, a prerequisite for tight junction formation. ZO-1 is concentrated at contact points of cells to create a barrier function seal between the cells [28]. Expression of ZO-1 protein varied between donors with low shear enhancing ZO-1 in donors 2 and 3 and high shear enhancing ZO-1 in donor 2 cells. While it is difficult to determine exactly how these shear stress rates affect barrier function it can be concluded that donor variations will influence the cells response to shear stress and formation of an intact barrier function. Perhaps a longer culture period with a more sophisticated experimental set up such as *trans*-epithelial resistance measurements could help to elucidate how shear stress affects the barrier function capability of the corneal epithelium.

A total of three human donors were used for this study, analysis of each donor separately was conducted due to the variation of ages between donors which influences gene and protein expression in LECs [29]. Other cell types have also been shown to have their therapeutic efficacy affected by donor age [30] including adipose derived stem cells [31] mesenchymal stem cells [32], hematopoietic stem cells transplantation [33] and for T-cell therapy specifically [34]. This study also showed that donor age affected LECs response to fluid flow. However, further factors which may affect donor response to fluid flow include gender, health, time from death to enucleation, storage time and cause of death which weren't considered in this study. In addition, the heterogeneity of the cultured cells could easily vary between donors. To more clearly identify if age is the factor that is influencing results, a much larger study with multiple donors from each age group would be required but due to the limited availability of tissue this was not possible for this study.

This data suggests that culturing LECs under low shear laminar flow rate would be optimum for upregulating stem cell markers in LECs under culture for transplantation. Additionally, culturing LECs under high shear conditions allow the cells to adopt a more stratified assembly, which may serve as an in-vitro model of the corneal epithelium to study.

## 5 Conclusion

This study has shown the mechanosensitivity of cultured corneal epithelial cells under fluid flow conditions significantly affects the cells adhesion, gene expression and stratification. Upregulation of TRPV4, demonstrates that the cells sense the fluidic shear forces and modulate their behaviour accordingly. Under high shear conditions the cells adopt a more stratified assembly, which may serve as an in-vitro model of the corneal epithelium to study. In addition to demonstrating mechanosensitivity, this work has shown that rather than using typical static culture, a new way of culturing these cells under shear stress produces a more conducive cellular phenotype. This may aid in higher success rates of transplantation while also aiding in our knowledge of the corneal epithelial mechanobiology. However, donor to donor variations and the heterogeneous nature of primary limbal epithelial cultures would need to be considered if using fluid shear to modulate cell behaviour.

## CRediT authorship contribution statement

**Sophia Masterton:** Data curation, Formal analysis, Investigation, Methodology, Writing – original draft, Writing – review & editing. **Mark Ahearne:** Conceptualization, Funding acquisition, Project administration, Supervision, Validation, Writing – review & editing.

## Declaration of competing interest

The authors declare the following financial interests/personal relationships which may be considered as potential competing interests:Mark Ahearne reports financial support was provided by European Research Council. Mark Ahearne reports financial support was provided by Irish Research Council. Sophia Masterton is currently employed by Eli Lilly and Company. This work was conducted independently of this employment, and Eli Lilly and Company had no role in the study design, data collection, analysis, interpretation, or manuscript preparation. If there are other authors, they declare that they have no known competing financial interests or personal relationships that could have appeared to influence the work reported in this paper.

## Data Availability

Data will be made available on request.
