# Supplementary material for: Influence of fluid shear stress on human limbal epithelial cells
Source: Biochem Biophys Rep. 2026 Jan 17;45:102453. doi: 10.1016/j.bbrep.2026.102453 (PMC12854039; doi:10.1016/j.bbrep.2026.102453)
Supplement: Multimedia component 1 [file mmc1.pdf]

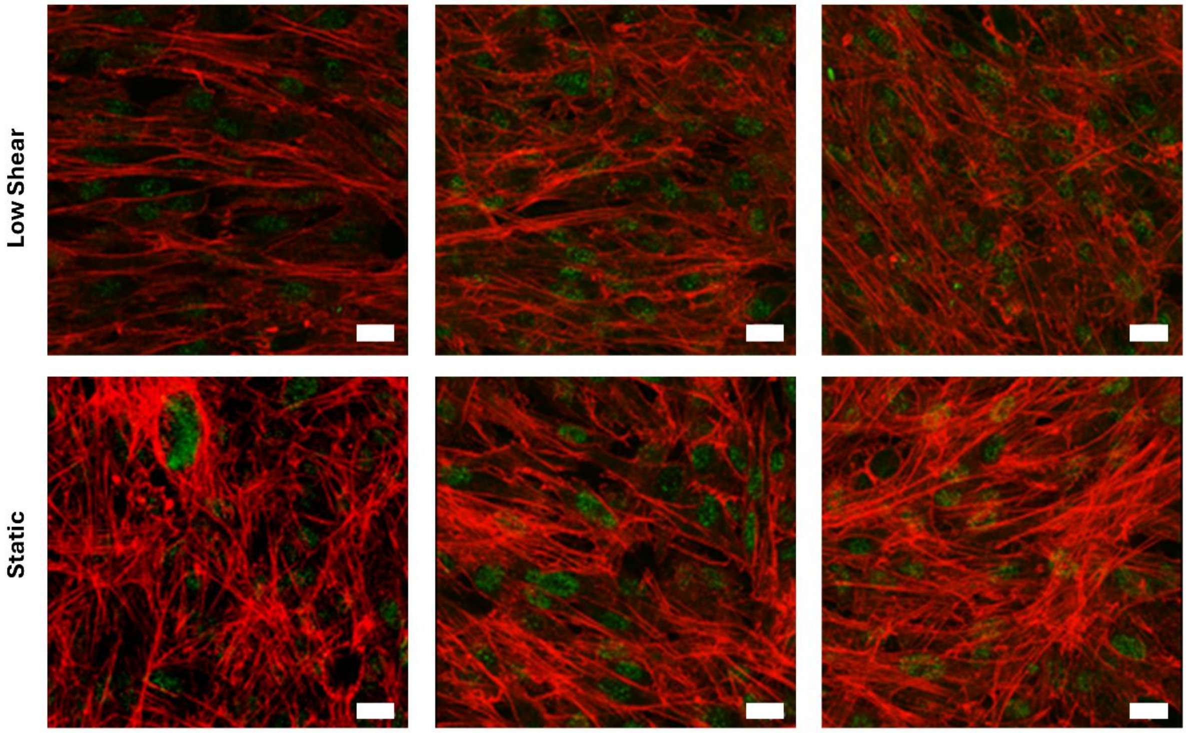

Figure S1: Immunocytochemical staining for NP63 in Donor 2. Nuclear localisation of NP63 after 1 day of low shear stress (top row) and under static conditions (bottom row) (Scale = 10 µm).

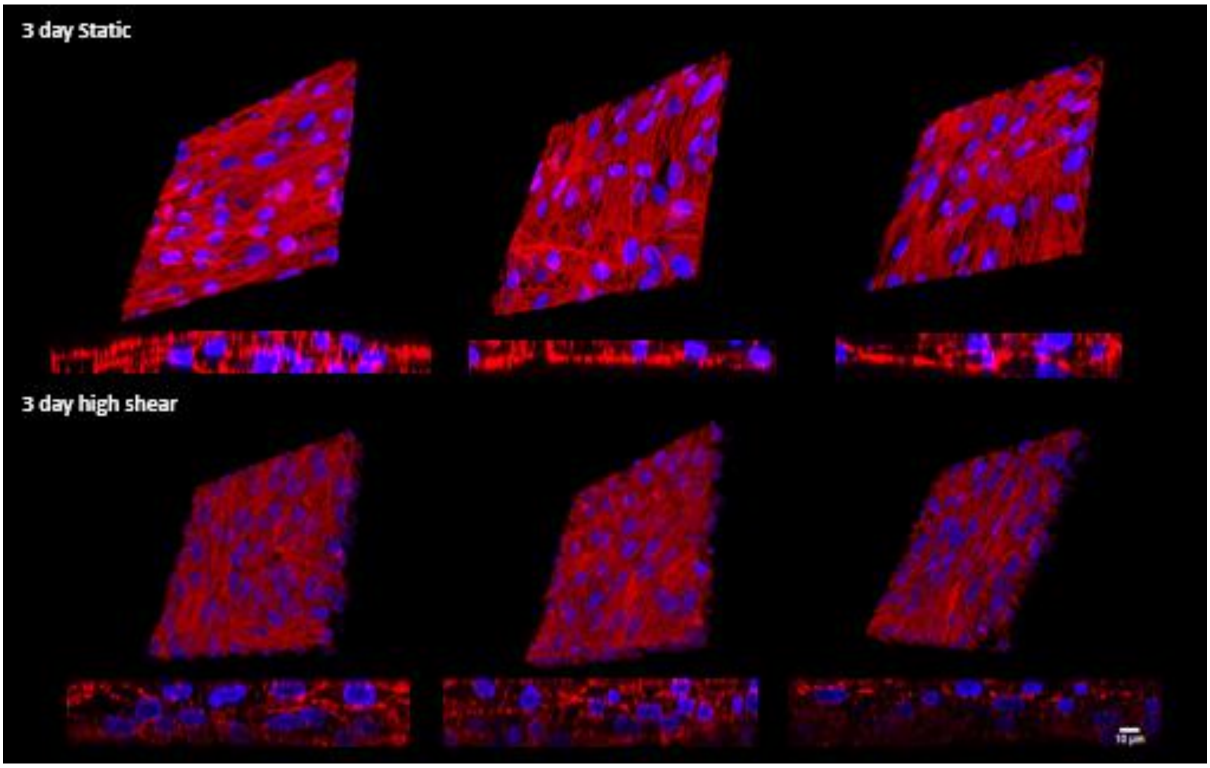

Figure S2: Immunocytochemical staining for cellular stratification: Maximum projection of Donor 3 after 3 days of static of high shear cell culture (Scale = 10 µm).
